# Risk factors for tuberculous empyema in pleural tuberculosis patients

**DOI:** 10.1038/s41598-019-56140-4

**Published:** 2019-12-20

**Authors:** Peng Wen, Min Wei, Chao Han, Yu He, Mao-Shui Wang

**Affiliations:** 1 Department of Respiratory Medicine, Shandong Provincial Chest Hospital, Shandong University, Jinan, China; 2 Department of Geriatrics, Shandong Mental Health Center, Jinan, China; 3 Department of Clinical Laboratory, First Affiliated Hospital of Guangxi Medical University, Nanning, China; 4 Department of Lab Medicine, Shandong Provincial Chest Hospital, Shandong University, Jinan, China

**Keywords:** Tuberculosis, Risk factors

## Abstract

Tuberculous empyema (TE) is associated with high mortality and morbidity. In the retrospective cohort study, we aimed to find risk factors for TE among pleural tuberculosis (TB) patients. Between July 2011 and September 2015, all culture-confirmed pleural TB patients (474 cases) were enrolled in our study. Empyema was defined as grossly purulent pleural fluid. Demographic and epidemiological data were collected for further analysis. Multivariate logistic regression analysis was used to evaluate risk factors of TE in pleural TB, age–adjusted odds ratio (OR) and 95% confidence interval (CI) were calculated to show the risk. The mean age was 35.7 ± 18.1 years old, males comprised 79.1% of the participants (375 cases). Forty-seven patients (9.9%) were multidrug-resistant TB (MDR-TB), 29 (6.1%) had retreatment TB, 26 (5.5%) had diabetes mellitus. The percentage of empyema patients was 8.9% (42 cases). Multivariate analysis revealed that male (adjusted OR = 4.431, 95% CI: 1.411, 13.919), pleural adenosine deaminase (ADA, >88 U/L) (adjusted OR = 3.367, 95% CI: 1.533, 7.395) and white blood cell (WBC, >9.52 10^9^/L) (adjusted OR = 5.763, 95% CI: 2.473, 13.431) were significant risk factors for empyema in pleural TB, while pulmonary TB (adjusted OR = 0.155, 95% CI: 0.072, 0.336) was the protective factor for the patients. TE remains a serious threat to public health in China. Male sex is a significant risk factor for TE while the presence of pulmonary TB is protective, and high levels of pleural ADA and WBC count could aid in early diagnosis of TE. This finding would help towards reducing the mortality and morbidity associated with TE.

## Introduction

Globally, tuberculosis (TB) remains one of the serious public health problems, especially in the high TB burden countries like China. According to WHO, in 2017, an estimated 10.0 million were newly diagnosed with TB worldwide, about 14% of them were extrapulmonary TB (EPTB), and 160 000 cases were multidrug-resistant TB (MDR-TB), which is defined as resistance to at least rifampicin and isoniazid^1^. Recently, a survey study in Tianjin, China demonstrated that pleural TB was the most common type of EPTB and accounted for two-thirds of all EPTB cases^2^. Therefore, it is thought that pleural TB may be frequent in high-incidence TB countries^3^. Despite treatment, pleural TB may progress to tuberculous empyema (TE), which can cause a chronic and fatal sequelae^4^.

TE is an uncommon form of pleural TB. It is characterized as a purulent infection occupying the pleural space and bacilli can be found in pleural effusion (PE)^5^. According to the American Thoracic Society, an empyema refers to a collection of pus in the pleural space and could be classified into three progressive stages, namely the exudative (I), fibrinopurulent (II) and organizing (III) phrases^6^. Over the past few decades, the incidence of TE has decreased significantly, but it still threatens public health^7^. Although this situation is changing with the development of medical treatment (such as potent anti-TB medication, punctures/drainage, and appropriate intrapleural antifibrinolytics), however, the increasing HIV epidemic would increase the risk of TB in developing countries^8,9^, and could reverse the trend. Moreover, for empyema, the mortality rate among HIV-infected patients is higher than that of non-infected patients (26.2% vs 5.1%)^10^.

Untreated or inadequately treated TE may result in a pleurocutaneous fistula (empyema necessitatis), chest wall mass, and rib and bone destruction^11^. Moreover, the development of TE is associated with high mortality and morbidity^6^. It is suggested that, in the UK, 20% of patients with empyema die, and approximately 20% require surgery to achieve cure within 1-year of initial infection^12,13^. Several factors, such as chronic respiratory diseases, diabetes mellitus, malignancies, immunosuppression, gastro-oesophageal reflux disease and alcohol and drug addiction, are found to increase the risk of progression to empyema^14^. However, risk factors for TE remains unclear. In the retrospective cohort study, we aimed to evaluate risk factors for TE among pleural TB patients.

## Materials and Methods

The retrospective study was performed at the Shandong Provincial Chest Hospital (SPCH), located in Jinan city in eastern China. SPCH is a provincial referral TB hospital of approximately 800 beds. Each year, about 500 patients with pleural TB are diagnosed, most of them are diagnosed clinically, not confirmed by culture or pathological examination. In Shandong Province, which has a population of 90 million, 40, 000 new TB cases are found annually and about 10% isolates are MDR-TB^15^. This study was approved by the SPCH Ethics Committees. Under Article 39.1 of ethics guidelines enacted by National Health and Family Planning Commission of the PRC (http://www.gov.cn/gongbao/content/2017/content_5227817.htm), this study was exempt from the need for written informed consent, as it used only secondary data.

Between July 2011 and September 2015, consecutive culture-confirmed pleural TB patients were enrolled in our study. Empyema was defined as grossly purulent pleural fluid^16^. Demographic and epidemiological data were collected using a questionnaire from electronic medical records, including sex, age, contact history of TB, smoking habit, treatment delays, underlying diseases and symptoms, and then were analyzed as described previously^17^.

*Mycobacterium tuberculosis* (*M.TB*) strains underwent drug susceptibility testing by using the absolute concentration method (isoniazid: 1 μg/ml, rifampicin: 50 μg/ml) on Lowenstein-Jensen medium^15^. PE samples obtained by thoracocentesis underwent biochemical and microbiological analysis. An automated chemistry analyzer (Advia 2400, Siemens Healthcare Diagnostics, Tokyo, Japan) was used to examine biochemical parameters, such as total protein, total bilirubin, glucose, lactate dehydrogenase (LDH) and adenosine deaminase (ADA). Hematological tests and flow cytometry analysis were performed on the EDTA whole blood, using a XT-1800i hematology analyzer (Sysmex Corporation, Kobe, Japan) and FACSCalibur Flow Cytometer (BD Biosciences, San Diego, USA), respectively.

Patient characteristics were summarized using means and standard deviations (or median and interquartile range) for continuous variables and counts/percentages for categorical variables. Categorical variables were compared using the χ2 test or Fisher exact test, and continuous variables compared using the Mann-Whitney *U* test or *t* test depending on the distribution of the data. Beyond descriptive statistics, associations between TE and clinical characteristics were analyzed by calculating the odds ratios (OR) and 95% confidence interval (CI), adjusted by age. Univariate logistic regression analysis was used to evaluate risk factors for TE, and significant variables (*P* < 0.1) were included in the multivariate logistic regression model. Prior to conducting multivariate logistic regression, continuous variables were transformed into categorical variables based on the cutoff points determined with receiver operating characteristic curve (ROC) analysis. Multivariate logistic regression analysis was then performed, including significant variables estimated in the univariate analysis or variables suggested in the literature. The Hosmer-Lemeshow goodness-of-fit test was performed to assess the overall fit of the model. A two-sided *P* value < 0.05 was considered significant for all analyses. Data analysis was carried out using SPSS 16.0 (IBM Corp., Armonk, United States).

## Results

### Characteristics of patients

Table 1 shows the demographic characteristics of the 474 participants of this study. All 474 pleural TB patients (Additional File 1) enrolled were culture-confirmed, and 42 of them (8.9%) were diagnosed as TE because of an accumulation of pus in the pleural space. The mean age was 35.7 ± 18.1 years old, males comprised 79.1% of the participants. Forty-seven patients (9.9%) were MDR-TB. Forty-nine (10.3%) patients had contact of TB history. Smokers constituted 51.3% of the participants. The delay period of treatment was 94.3 ± 237.6 days. Among them, 344 (72.6%) participants had pulmonary TB, 70 (14.8%) had EPTB (excluding pleural TB), 29 (6.1%) had retreatment TB, 26 (5.5%) had diabetes mellitus, 4 (0.8%) had milliary TB. Cough (71.1%) was the most commonly reported symptom. Fever (70.7%), dyspnea (51.5%), chest pain (41.4%), sputum production (37.6%), and fatigue (8.2%) were the other symptoms. Chest X-ray revealed that 56.8% of the effusions were on the right, 33.8% on the left and 9.5% on the both.

**Table 1:** Univariate analysis of the demographic data associated with TE in pleural TB.

	Non-TE (n)	TE (n)	Total	OR (95%CI)	P value
N	432 (91.1%)	42 (8.9%)	474		
Sex (male)	337 (78.0%)	38 (90.5%)	375 (79.1%)	2.678 (0.932, 7.692)	0.067
Age (years)	36.3 ± 18.5	29.1 ± 12.2	35.7 ± 18.1	0.973 (0.951, 0.995)	0.016
MDR-TB	42 (9.7%)	5 (11.9%)	47 (9.9%)		0.652
Contact history of TB	47 (10.9%)	2 (4.8%)	49 (10.3%)		0.228
Smoking habit (pack-years)	7.67 ± 16.76	2.83 ± 7.25	7.24 ± 16.20	0.410 (0.096, 1.750)	0.082
Treatment delays (days)	30 (14, 60)	60 (30, 150)	30 (15, 90)		0.210
Comorbidity					
Pulmonary TB	326 (75.5%)	18 (42.9%)	344 (72.6%)	0.244 (0.127, 0.467)	0.000
Extrapulmonary TB	64 (14.8%)	6 (14.3%)	70 (14.8%)		0.926
Retreatment TB	24 (5.6%)	5 (11.9%)	29 (6.1%)		0.110
Diabetes mellitus	25 (5.8%)	1 (2.4%)	26 (5.5%)		0.371
Milliary TB	4 (0.9%)	0 (0%)	4 (0.8%)		0.999
Symptoms					
Cough	309 (71.5%)	28 (66.7%)	337 (71.1%)		0.508
Fever	303 (70.1%)	32 (76.2%)	335 (70.7%)		0.412
Dyspnea	216 (50.0%)	28 (66.7%)	244 (51.5%)	2.000 (1.025, 3.903)	0.042
Chest pain	184 (42.6%)	12 (28.6%)	196 (41.4%)		0.082
Sputum production	165 (38.2%)	13 (31.0%)	178 (37.6%)		0.356
Fatigue	37 (8.6%)	2 (4.8%)	39 (8.2%)		0.399
Effusion sites					
Right	238 (55.1%)	31 (73.8%)	269 (56.8%)	2.297 (1.125, 4.689)	0.022
Left	151 (35.0%)	9 (21.4%)	160 (33.8%)		0.082
Both	43 (10.0%)	2 (4.8%)	45 (9.5%)		0.285

TE, tuberculous empyema; TB, tuberculosis; OR, odds ratio; CI, confidence interval; MDR-TB, multidrug-resistant tuberculosis.

The results of PE biochemical tests, flow cytometry analysis and hematological tests were also summarized in Table 2 and Supplementary Table 1. For comparisons between groups in terms of continuous variables, Mann-whitney U tests showed that differences in glucose (*P* < 0.001), LDH (*P* < 0.05), ADA (*P* < 0.05), red blood cell count (*P* < 0.01), hemoglobin (*P* < 0.01), hematocrit (*P* < 0.01) and erythrocyte sedimentation rate (*P* < 0.05) were significant when comparing the two groups. The *t* tests showed that age (*P* < 0.05) and white blood cell count (WBC, *P* < 0.05) were significantly different between the two groups. The other continuous variables did not reach significance (all *P* > 0.05). For dichotomous variables, chi-square analysis of these data showed that the two groups were significantly different in terms of pulmonary TB (*P* < 0.001), dyspnea (*P* < 0.05) and effusion site (right, *P* < 0.05). The other categorical variables did not reach significance (all *P* > 0.05).

**Table 2:** Univariate analysis of the laboratory data associated with TE in pleural TB.

	Non-TE (n)	TE (n)	Total	OR (95%CI)	P value
N	432 (91.1%)	42 (8.9%)	474		
Biochemical analysis (PE)					
Total protein (g/L)	46.1 ± 13.8	45.7 ± 12.6	46.0 ± 13.7		0.880
Total bilirubin (mmol/L)	10.1 ± 7.1	9.2 ± 7.5	10.0 ± 7.1		0.423
Glucose (mmol/L)	4.18 (2.65, 5.75)	2.90 (0.22, 4.62)	4.10 (2.37, 5.69)	0.802 (0.697, 0.922)	0.002
LDH (U/L)	648 (383, 984)	812 (410, 2652)	654 (385, 1015)	1.000 (1.000, 1.000)	0.001
ADA (U/L)	52 (40, 67)	55 (45, 122)	52 (41, 70)	1.009 (1.004, 1.013)	0.000
Amylase (U/L)	30.1 ± 14.6	29.2 ± 11.8	30.0 ± 14.3		0.688
Hematological tests					
WBC (10^9^/L)	6.99 ± 2.67	8.13 ± 3.63	7.08 ± 2.78	1.123 (1.025, 1.232)	0.013
Red blood cell (10^12^/L)	4.36 (3.98, 4.71)	4.66 (4.39, 5.07)	4.39 (4.00, 4.73)	2.385 (1.332, 4.270)	0.003
Hemoglobin (g/L)	125 (113, 136)	135 (122, 144)	126 (114, 138)	1.023 (1.005, 1.041)	0.014
Hematocrit (%)	37.5 (34.3, 40.4)	39.3 (37.7, 43.2)	37.7 (34.4, 40.8)	1.098 (1.026, 1.176)	0.007
Erythrocyte sedimentation rate (mm/h)	44.0 (27.0, 67.0)	36.0 (18.0, 48.0)	43.0 (27.0, 66.0)	0.985 (0.972, 0.998)	0.027

TE, tuberculous empyema; TB, tuberculosis; OR, odds ratio; CI, confidence interval; PE, pleural effusion; LDH, lactate dehydrogenase; ADA, adenosine deaminase; WBC, white blood cell.

### Univariate and multivariate analysis

Tables 1 and 2 shows the univariate analysis of risk factors, comparing patients with empyema with patients without empyema. The presence of empyema was associated with age (OR = 0.973, 95% CI: 0.951, 0.995), pulmonary TB (OR = 0.244, 95% CI: 0.127, 0.467), dyspnea (OR = 2.000, 95% CI: 1.025, 3.903), effusion site (right, OR = 2.297, 95% CI: 1.125, 4.689), pleural biochemical tests (glucose (OR = 0.802, 95% CI: 0.697, 0.922), LDH (OR = 1.000, 95% CI: 1.000, 1.000) and ADA (OR = 1.009, 95% CI: 1.004, 1.013)) and hematological tests (WBC (OR = 1.123, 95% CI: 1.025, 1.232), red blood cell (OR = 2.385, 95% CI: 1.332, 4.270), hemoglobin (OR = 1.023, 95% CI: 1.005, 1.041), hematocrit (OR = 1.098, 95% CI: 1.026, 1.176) and erythrocyte sedimentation rate (OR = 0.985, 95% CI: 0.972, 0.998)) (all *P* < 0.05).

To make the results as readily understandable as possible, continuous variables were converted into dichotomous categorical variables based on the cutoff points determined with ROC analysis, and the corresponding optimal cutoff values were 88 U/L and 9.52 10^9^/L for ADA or WBC, respectively. Further multivariate analysis (Hosmer–Lemeshow goodness-of-fit test: χ^2^ = 2.329, df = 5, P = 0.802) revealed that male (adjusted OR = 4.431, 95% CI: 1.411, 13.919), pleural ADA (>88 U/L) (adjusted OR = 3.367, 95% CI: 1.533, 7.395) and WBC (>9.52 10^9^/L) (adjusted OR = 5.763, 95% CI: 2.473, 13.431) were significant risk factors for empyema in pleural TB, while pulmonary TB (adjusted OR = 0.155, 95% CI: 0.072, 0.336) was the protective factor for the patients (Table 3, Additional File 2).

**Table 3:** Age-adjusted OR for risk factors associated with TE in pleural TB.

	Age-adjusted OR	P value
Sex (male)	4.431 (1.411, 13.919)	0.011
Pulmonary TB	0.155 (0.072, 0.336)	<0.001
ADA (>88 U/L)	3.367 (1.533, 7.395)	0.002
WBC (>9.52 10^9^/L)	5.763 (2.473, 13.431)	<0.001

TE, tuberculous empyema; TB, tuberculosis; OR, odds ratio; CI, confidence interval; ADA, adenosine deaminase; WBC, white blood cell.

## Discussion

Currently, the pathogenesis of empyema is not well understood^18^. TE, is less common and represents an infection of the pleural space by *M.TB* that provokes the accumulation of purulent pleural fluid^19^. TE usually occurs in younger and middle-aged patients^20^. However, the accurate incidence has not been properly evaluated recently. In a previous study, TE has been reported that accounts for 3%–6% of all cases of empyema in South Korea^21^. In this study, we conducted a retrospective cohort study to evaluate the risk factors of TE in pleural TB patients. To our best knowledge, this is the first report detailing risk factors for the incidence of TE among pleural TB patients in China.

Our results indicated that pulmonary TB is a protective factor for TE. TE usually develops due to chronic pulmonary TB^22^, and rarely as a result of untreated tuberculous pleural effusion (TPE)^23^. Prior to the diagnosis of TE, patients often have suffered from chronic pulmonary TB for more than 10 years^6^. This is due to the fact that most of TE patients are asymptomatic^24^. Nevertheless, sputum culture positive for *M.TB* is also considered as a gold standard criteria for TPE diagnosis^25^. Therefore, the TE secondary to pulmonary TB would be more easily detected, because of the additional specimen (sputum) examined. Then, anti-TB drugs would be given timely, in other words, this may have a positive effect on the prognosis of TE patients. In addition, Ornstein GG *et al*. suggested that, when the underlying pulmonary TB is under control, the prognosis of TE is good; nevertheless, when the associated pulmonary TB not under control, the prognosis is not good^26^. This also indicates that an increased association between pulmonary TB and TE outcomes is plausible.

In the study, we found that male sex was a risk factor for TE. A possible explanation for it is that sex imbalance exists in TPEs^27,28^. Das DK *et al*. analyzed the sex distribution of TPE patients included in the previous published literatures, and most of studies (3/20) showed that female patients constituted the majority of TPEs (>50%)^28^. In the United States, from 1993 through 2003, 7,549 cases of pleural TB were reported, males comprised 67% and females 33% of the patient population. A similar result was reported in a retrospective study of 254 pleural TB patients^29^.

High levels of pleural ADA and peripheral WBC count predict increased TE risk. The findings suggest that the two biomarkers may reveal the progression of TE. Several reports have discovered the diagnostic role of the two in patients with empyma. Li R *et al*. reported that PE ADA could be used as an alternative biomarker for early and quick discrimination of Gram-negative from Gram-positive bacterial infections of the pleural space, which is useful for the selection of antibiotics^30^. A retrospective study conducted by Porcel JM *et al*. concluded that a high level of ADA is a general characteristic of lymphocytic and neutrophilic TB effusions, and an extremely high ADA activity is usually considered in empyema or lymphoma^31^. For the treatment of empyema, the stage of its development is the main parameter to be considered. A variety of treatments, including appropriate antibiotic therapy, pleural drainage, decortications, thoracotomy and video-assisted thoracoscopic surgery, must be selected and combined together in the most appropriate way with optimal timing^32^. However, currently there is no biomarker available that would help to choose appropriate interventions^33^. Thus, careful consideration should be given to the indications of each therapy for critical patients. In the next study, we would examine whether the pleural ADA level and peripheral WBC count could help to identify stage II–III empyema thoracis.

The study was conducted in a large population, and the findings represent a valuable contribution to the field. However, several limitations in the study are worth noting. First, retrospective nature could not control patients’ baseline characteristics, so the results might be subject to selection bias. Second, as you know, the stage of empyema is considered as a main factor for selection of appropriate treatment approach. If an association between empyema stages and these clinical-pathological characteristics is observed, it may aid to control the development of TE. However, we failed to investigate it, this was because of the lack of information on the empyema stage of each patient. Third, because of a single-center design, the findings may be limited which may not accurately reflect the general characteristics of TE in China. In addition, although several risk factors were identified, further analysis must be performed to validate our findings.

## Conclusions

Our study found that male sex is a significant risk factor for TE while the presence of pulmonary TB is protective, with high levels of pleural ADA and WBC count could aid in early diagnosis of TE. Given the high proportion of TE in pleural TB was reported, TE remains a serious threat to public health in China.

## Supplementary information

Supplementary Table 1

Additional file 1

Additional file 2
